# Sclerosing angiomatoid nodular transformation (SANT) of the spleen: a case report

**DOI:** 10.1093/omcr/omae158

**Published:** 2024-12-28

**Authors:** Sotiris Loizides, Paraskevi Samouti, George Tsironis, Eleni Xenophontos, Demetrios Papamichael, Vassilios Vassiliou

**Affiliations:** Department of Medical Oncology, Bank of Cyprus Oncology Center, 32 Acropoleos Avenue, Nicosia 2011, Cyprus; Department of Medical Oncology, Bank of Cyprus Oncology Center, 32 Acropoleos Avenue, Nicosia 2011, Cyprus; Department of Radiation Oncology, Bank of Cyprus Oncology Center, 32 Acropoleos Avenue, Nicosia 2011, Cyprus; Department of Medical Oncology, Bank of Cyprus Oncology Center, 32 Acropoleos Avenue, Nicosia 2011, Cyprus; Department of Medical Oncology, Bank of Cyprus Oncology Center, 32 Acropoleos Avenue, Nicosia 2011, Cyprus; Medical School, University of Nicosia, 46 Makedonitissas Avenue, Nicosia 2417, Cyprus; Department of Medical Oncology, Bank of Cyprus Oncology Center, 32 Acropoleos Avenue, Nicosia 2011, Cyprus; Department of Radiation Oncology, Bank of Cyprus Oncology Center, 32 Acropoleos Avenue, Nicosia 2011, Cyprus

**Keywords:** sclerosing angiomatoid nodular transformation, splenic lesion, splenectomy, case report, rectal cancer

## Abstract

SANT is a rare, non-lymphoid, benign entity, originating from the red pulp of the spleen. It is characterized by the presence of vascular nodules surrounded by a stroma of collagen fibers. It was introduced as a distinct disease entity by Martel et al in 2004, after the histopathological examination of 25 cases. Symptoms are unspecific, and in most cases, it is incidentally diagnosed, mostly in patients who undergo imaging for other underlying conditions. Moreover, radiological findings are usually inconclusive, and a definite diagnosis is established through histopathological examination. We herein report the case of a 76-year-old female diagnosed with stage I rectal cancer who was subsequently diagnosed with a splenic lesion after a Computed Tomography (CT) scan that was performed for follow-up purposes. Different imaging modalities were employed for further assessment; however, findings were inconclusive and the possibility of metastatic disease could not be excluded. The patient was referred for splenectomy and the diagnosis of SANT was established through histopathological examination.

## Introduction

Sclerosing angiomatoid nodular transformation (SANT) of the spleen represents a rare benign vascular lesion, with an incidence below 1%. Although it has been first reported by Krishnan in 1993, the term was established by Martel et al in 2004 [1]. Despite the fact that the pathophysiology of the disease remains uncertain, Epstein–Barr virus (EBV) and immunoglobulin IgG4-related sclerosing disease were shown to be involved in the development of SANT. The diagnosis remains challenging due to the absence of specific clinical and radiological features. In most cases, it is incidentally detected in patients who undergo routine follow-up imaging for different pathologies, as in the reported case, and may be misdiagnosed as metastases. In fact, when an imaging modality (e.g. CT) detects a splenic lesion, hemangioma, hamartoma, inflammatory pseudotumor, lymphoma, angiosarcoma, littoral angioma and metastases should be considered in the differential diagnosis [2]. The definite diagnosis is established through histopathological examination.

## Case report

**Figure 1:**
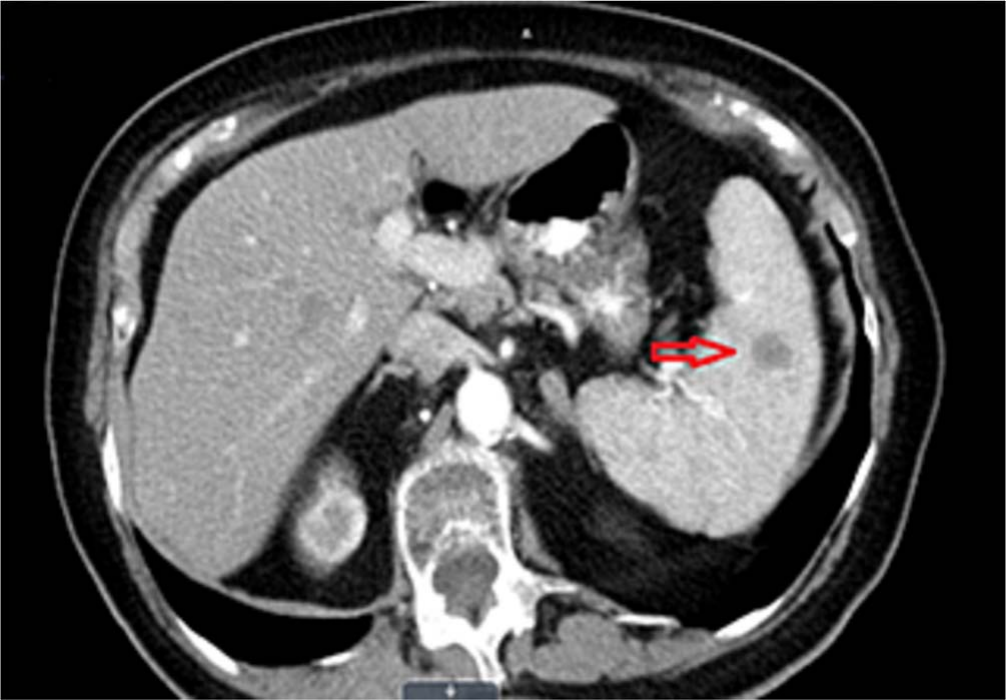
Transverse CT scan plane depicting hypodense splenic lesion (arrow).

**Figure 2:**
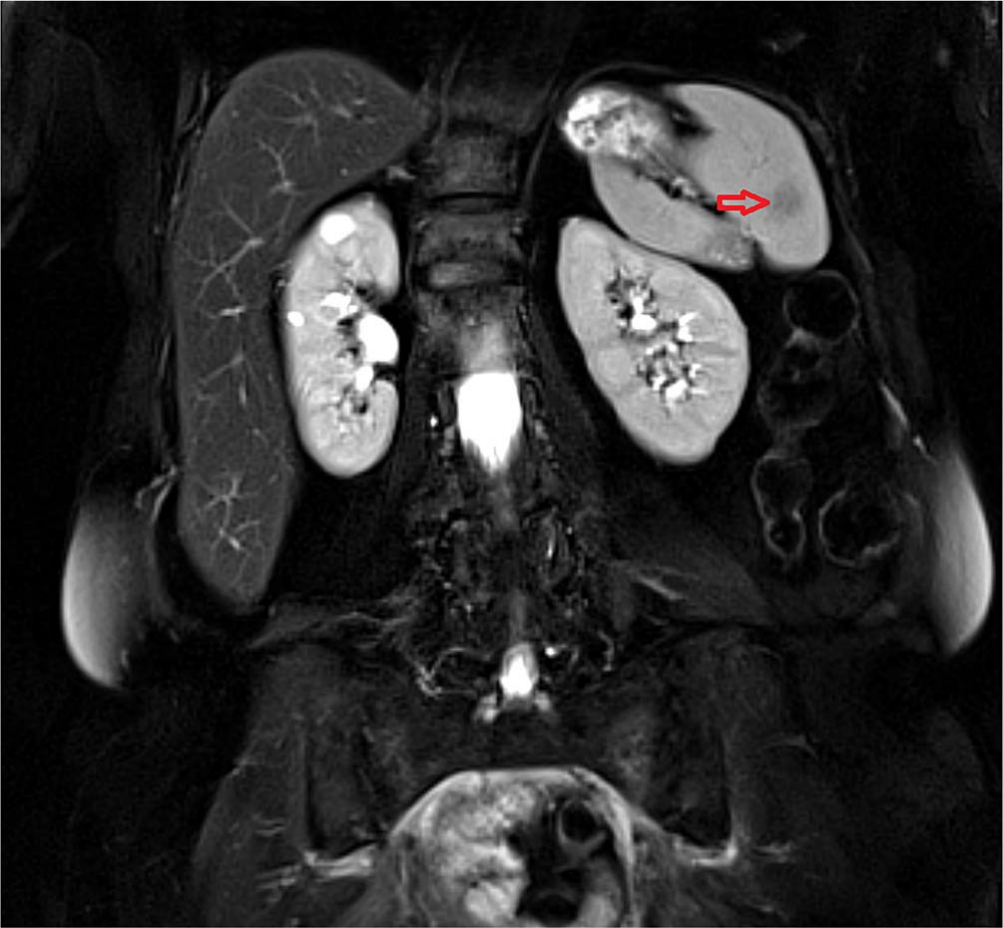
Coronal MRI plane demonstrating low signal intensity splenic lesion (arrow). T2-fat suppression image.

We report a case of a 76-year-old woman with a locally advanced mid-rectal adenocarcinoma who was operated after long-course chemoradiotherapy (CRT) in December 2019, (ypT2N0(0/14), stage I, AJCC 8^th^ edition) and was put on follow-up with CT scan surveillance every 6 months. Almost 2 years after surgery in September 2021, a CT scan revealed a new splenic lesion that was highly suspicious for metastasis. The lesion was hypodense and measured 12 mm in maximum diameter (Fig. 1). To further clarify the nature of the finding, an abdominal ultrasound was performed, also suggesting spleen metastasis. The case was discussed at the local Multidisciplinary team (MDT) meeting for gastrointestinal cancers and was decided to refer the patient for an 18F-fluoro-2-deoxyglucose (18-FDG) Positron Emission Tomography (PET) scan. This scan demonstrated no FDG uptake by the splenic lesion, however, a high Standardized Uptake Value (SUV = 7.2) was noted, at site of surgery suggesting local recurrence. Magnetic Resonance Imaging (MRI) of the abdomen and pelvis followed, with the scope to further evaluate the above findings. MRI showed a low signal intensity (T2 sequence) lesion within the spleen compatible with metastasis (Fig. 2), whereas the findings in the anastomosis were suggesting local recurrence or inflammation. Subsequently, the patient underwent a colonoscopy, with no signs of local relapse and biopsies taken from the anastomosis were compatible with inflammatory changes. The case was discussed anew at our MDT, and it was decided to refer the patient for splenectomy since this would be both diagnostic and therapeutic (local treatment for a single metastasis).

**Figure 3:**
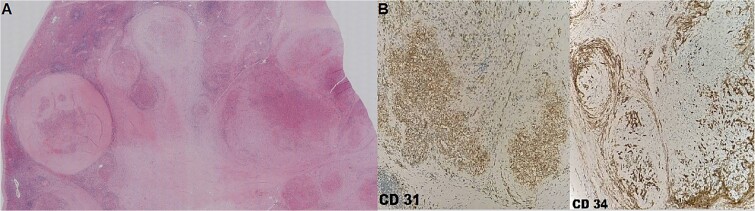
Histopathological examination of the lesion. (**A**) Multinodular lesion, surrounded by dense collagen stroma (Hematoxylin–eosin staining, magnification ×2). (**B**) The capillaries show strong immunoreactivity for both CD31 and CD34 (Immunohistochemical staining, magnification ×10).

The patient underwent splenectomy uneventfully in December 2021 with an excellent post-operative recovery. The histopathological examination revealed a solitary circumscribed, stellate mass of a maximum diameter of 1.6 cm. Microscopically this lesion consisted of multiple angiomatoid nodules that contained slit-like vascular channels surrounded by a stroma of dense collagen (fibrosis). Immunohistochemical examination showed that capillaries were positive for CD31 and CD34 and small veins were positive for CD31. Macrophages within the lesion were positive for CD68. The above findings were consistent with SANT (Fig. 3). No signs of neoplasia were noted. At the time of publication, the patient is in an excellent clinical condition and on regular follow-up.

## Discussion

SANT is a rare non-lymphoid splenic tumor. Even though it was noted by Krishnan et al in 1993 as “cord capillary hemangioma”, it was established as a distinct entity by Martel et al in 2004 [1]. Based on the histopathological examination of 25 cases, it was deduced that it is a benign vascular lesion originating from the red pulp of the spleen.

SANT is not associated with any specific symptoms or signs. As with most splenic lesions, it is discovered incidentally after imaging for different unrelated conditions. SANT is mostly detected in the context of malignancy. Reported symptoms are abdominal pain, weight loss, fatigue, fever, and palpable mass. Moreover, 4 cases were related to thrombocytopenia. Interestingly, SANT predominantly occurs in women [3–5].

The exact pathogenesis of SANT is still unclear. It is postulated that it is the last step of a cataract of processes in the background of an inflammatory pseudotumor, distortion of the red pulp and excessive non-neoplastic stromal proliferation [5]. Additionally, it is associated with EBV infection [6], and immunoglobulin IgG4-related sclerosing disease. Some authors have reported a significantly higher count of IgG4+ plasma cells and an increased IgG4/IgG ratio in affected patients [7].

Radiological diagnosis of SANT is challenging, especially in cases with a known malignancy. In such patients, new splenic lesions raise the possibility of metastasis that rarely affects the organ. In scientific literature, various radiological characteristics have been described with different imaging modalities to correctly diagnose SANT [8]. The combination of different imaging modalities increases the possibility for a non-interventional diagnosis, avoiding biopsies or splenectomy. In the meta-analysis by Aziret et al 62 out of 230 examined patients had a definite diagnosis of SANT preoperatively [3].

The gold standard for SANT diagnosis is the histopathological examination of the lesion. Macroscopically, it is a well circumscribed mass with multiple nodules separated by fibrotic columns. Microscopically, it illustrates a multinodular growth pattern and fissure-like vascular spaces surrounded by a plethora of collagen fibers and inflammatory cells comprising of lymphocytes, plasma cells and macrophages. The benign characteristics are based also on the absence of nuclear atypia and the rarity of mitotic figures and necrosis. On immunohistochemical staining, three distinct types of vascular structures are recognized, capillaries, sinusoids and small vessels. Capillaries are CD34 and CD31 positive, sinusoids are CD8 and CD31 positive whereas small vessels are CD31 positive [9].

Splenectomy can be both diagnostic and curative. Open splenectomy or minimally invasive techniques can be used. Potential complications after splenectomy include susceptibility to infections from encapsulated bacteria, thromboembolic episodes, suture granuloma, fluid collection and multi-organ failure. Considering the pros and cons of splenectomy Tseg et al suggested that surgical option for a benign splenic lesion should be considered in cases of symptomatic relief, splenic rupture, cytopenia due to spleen sequestration and in cases where imaging cannot exclude malignancy [10]. Considering the difficulty in establishing the diagnosis through radiological imaging, the decision for splenectomy should be taken after MDT discussion and after fully informing patients about potential complications.

## Conclusion

SANT is a quite rare benign splenic lesion and encounters for <1% of all splenic masses. Its diagnosis is challenging due to the rarity of the lesion as well as the lack of specific radiographic features. Most of the times SANT is detected incidentally in the context of investigation of other diseases such as cancer, likewise in our case. Diagnosis is established after splenectomy and histopathological examination of the lesion.
